# CAF promotes chemoresistance through NRP2 in gastric cancer

**DOI:** 10.1007/s10120-021-01270-w

**Published:** 2021-11-26

**Authors:** Yanpeng Yang, Yongchen Ma, Shen Yan, Pengyuan Wang, Jianwen Hu, Shanwen Chen, Jing Zhu, Jingui Wang, Guowei Chen, Yucun Liu

**Affiliations:** 1 Department of Gastroenterology, Peking University First Hospital, No. 8 Xishiku Street, Xicheng District, Beijing, China; 2 Department of Endoscopic Center, Peking University First Hospital, Beijing, China; 3 Department of Academy of Medical Engineering and Translational Medicine, Tianjin University, Tianjin, China

**Keywords:** Cancer-associated fibroblasts, Gastric cancer, NRP2, Chemoresistance

## Abstract

**Background:**

Fibroblasts are the predominant cell type in the stroma of tumor, and cancer-associated fibroblasts (CAFs) promote cancer chemoresistance by secreting various bioactive molecules. However, the differential expression between CAFs and normal fibroblasts (NFs) and how can CAFs uniquely impact cancer cells are still unexplored.

**Methods:**

Primary CAFs and NFs were cultured from gastric cancer specimens, and their variant expression was analyzed by RNA-sequencing. Chemoresistance was evaluated by measuring cell viability, apoptosis, and 3D-coculture techniques.

**Results:**

CAFs were isolated from gastric cancers and defined by specific cell-surface markers. CAFs decreased the sensitivity of gastric cancer cells to 5-FU. RNA-sequencing showed that CAFs expressed a higher level of NRP2 than NFs. And the high expression of NRP2 was correlated with worse oncological outcomes in gastric cancer patients. Further study showed that the knockdown of NRP2 eradicated the resistance to 5-FU. And the secretion of stromal cell-derived factor-1 (SDF-1) was reduced following NRP2 knockdown. Furthermore, we found that the increased sensitivity to 5-FU was induced by DNA damage. And this process was mediated by predominant effectors of the Hippo pathway, YAP/TAZ.

**Conclusions:**

The present study indicated that CAFs within gastric cancers promote chemoresistance through the expression of NRP2. The secretion of SDF-1 that mediated by VEGF/NRP2 signaling in CAFs and the activation of Hippo pathway in cancer cells in large part participated in this project.

**Supplementary Information:**

The online version contains supplementary material available at 10.1007/s10120-021-01270-w.

## Background

Tumorigenesis and progression are complex processes containing complicated cross-talk between malignant cells and their surrounding stromal components, including cellular and acellular elements known as tumor microenvironment (TME). Fibroblasts are not only the major cell types within the stroma, but also the predominant source of acellular tissue containing soluble molecules and the extracellular matrix [1]. Scientists discovered that neighbor suppression is the specific function of normal fibroblasts (NFs), which can inhibit the progress of adjacent abnormal cells [2–4]. And several reports have described that the inhibition of malignant cells by NFs depends on directly contact and the secretion of soluble factors [5–8]. However, fibroblasts can switch from suppressors to tumor promoters upon various stimuli, which are called cancer-associated fibroblasts (CAFs) [9, 10]. CAFs can be identified through a series of markers such as vimentin, fibroblast-associated protein (FAP), fibroblast-specific protein 1 (FSP1), and alpha-smooth muscle actin (α-SMA) [11]. Multiple reports emphasized the contribution of CAFs to cancer initiation, growth, metastasis, and therapy resistance [12–18].

Gastric cancer is the fifth most commonly diagnosed carcinoma worldwide, and there are about 1,000,000 new cases in 2020 [19]. Despite advances in cytotoxic and targeted drugs, only a fraction of patients will benefit from them [20]. It has been demonstrated that CAFs confer resistance to cancer treatments via diverse pathways, like reduced drug delivery and anti-apoptosis signaling pathway [21]. However, studies focusing on CAFs in gastric cancer are in the bud compared with breast and pancreatic cancers.

Neuropilins (NRPs) are transmembrane glycoproteins and there are two NRPs expressed in human beings. NRP1 and NRP2 exhibit 44% identity at the amino acid level, and they contain four distinct extracellular domains that mediate ligand binding and a short cytoplasmic domain that lacks known activity [22, 23]. The critical finding of NRP2 is that it can function as the receptor of vascular endothelial growth factor (VEGF). This seminal finding launched studies that plan to understand their contributions to tumor biology [24]. Until now, multiple studies had recognized the importance of VEGF/NRP2 signaling to the behavior of tumor initiation and resistance to therapies [25, 26]. However, the function of NRP2 in CAFs is ambiguous.

In this study, we identified distinctly different expressed RNAs between NFs and CAFs of gastric cancer by RNA-sequencing. And after bioinformatic analysis, we found that NRP2 was recurrently upregulated in the nine CAF strains compared with matched NFs. Our results revealed that CAFs within gastric cancers promote chemoresistance through the expression of NRP2. The secretion of SDF-1 that mediated by VEGF/NRP2 signaling in CAFs and the activation of Hippo pathway in cancer cells in large part participated in this project.

## Methods

### Primary cell culture

Primary cancer-associated fibroblasts (CAFs) were isolated from advanced gastric adenocarcinoma samples obtained from surgery. Normal fibroblasts (NFs) were collected from normal gastric tissue of these surgery patients. Clinical characteristics of included patients were demonstrated in Supplementary file 1. All these cancers or normal samples were identified by pathology. Briefly, tissues were cut into pieces as small as possible, followed by bacterium eradication using 1% Penicillin–Streptomycin Solution (Gibco, USA) and 0.4% Normocin (Invivogen, France). Then, tissues were digested by 1 mg/mL collagenase type I (Invitrogen, USA) at 37 °C with shaking for 1.5–2 h. Thereafter, the dissociated tissues were collected by centrifuge at 1000 rpm for 5 min. Tissues were suspended by DMEM (Gibco, USA) with 20% FBS (Gibco, USA), and the stromal cell-enriched supernatants were separated to the culture bottle. And undigested tissues were collected to another bottle. Then, fibroblasts were incubated in DMEM with 20% FBS and validated by immunofluorescent staining and western blot. All specimens were collected from the patients with informed consent, and our research was approved by the internal review and ethics boards of Peking University First Hospital.

### RNA isolation, library preparation, and sequencing

Using TRIzol reagent, total RNA was extracted from tissues and cultured cells. RNA concentration was gauged by a Qubit^®^ RNA Assay Kit in Qubit^®^ 2.0 Fluorometer (Life Technologies, USA). Each sample extracted 20 ng RNA for the RNA sample preparations to be used as input material. Ribosomal RNA was extracted by Epicentre Ribo-zero™ rRNA Removal Kit, and ethanol precipitation was used to clean up the rRNA-free residue. Sequencing libraries were generated using the rRNA-depleted RNA by NEBNext^®^ Ultra™ Directional RNA Library Prep Kit for Illumina^®^ (NEB, USA) according to the manufacturer’s recommendations. The libraries were sequenced on an Illumina Hiseq 2500 platform and 125 bp paired-end reads were generated.

### Western blotting

The protein expression mentioned in our study was assessed by western blot analysis. Protein was blocked with 5% fat-free dried milk and incubated with anti-FAP (1:1000, CST), anti-Vimentin (1:1000, CST), anti-α-SMA (1:1000, CST), anti-NRP2 (1:1000, Abcam), anti-γH2AX (1:1000, Abcam), anti-YAP/TAZ (1:1000, CST), anti-SDF-1 (1:1000, Abcam), and anti-GAPDH (1:1000, CST) antibodies, respectively.

### Immunofluorescence

Cells were seeded on the coated coverslips. Cells were fixed with 4% paraformaldehyde and then permeabilized with 0.01% Triton X-100. Then, cells were treated with anti-FAP (1:100, CST), anti-Vimentin (1:100, CST), anti-α-SMA (1:100, CST), anti-γH2AX (1:100, Abcam), and anti-SDF-1(1:100, Abcam), and incubated with Alexa Fluor 488 goat anti-rabbit IgG. The nucleus was stained with DAPI.

### qPCR

Total RNA was isolated from cells using TRIzol reagent (ThermoFisher Scientific, USA), and the concentration of RNA was measured by the absorbance at 260 and 280 nm. M-MLV Reverse Transcriptase (ThermoFisher Scientific, USA) was used for the reverse transcription of RNA into cDNA. Quantitative real-time PCR was performed using SYBR Green (ThermoFisher Scientific, USA) according to the instructions, and the assays were carried out in the LightCycler480 system.

### Three-dimensional (3D) cell coculture and tumor sphere formation

To simulate the in vivo stereo structure, Perfecta3D plates (Sigma, USA) were used for the 3D coculture of CAFs and gastric cancer cells. Equal numbers of infected CAFs and SGC7901/BGC823 cells labeled by mScarlet were mixed and 50 μL of the suspension was added to each plate well. When challenged by drugs, cells were treated with 5-FU (200 μM, 6 μM for SGC7901 and BGC823, respectively). The sphere was harvested in the receiving plates.

### Cell survival assays

SGC7901 and BGC823 cells were added in 96-well plates in triplicates and challenged with increasing concentrations of 5-FU (dissolved by CM and normal medium, respectively) for 72 h. Then, the cell survival was detected using Cell Counting Kit-8 regents (Selleck, USA).

### Apoptosis assays

Cells were treated with 5-FU (250 μM,3 μM for SGC7901 and BGC823, respectively) for 48 h. Apoptosis was determined using Annexin V Apoptosis Detection Kit (BD, USA). After harvest, cells were washed with 100 μL of binding buffer, and then stained with 5 μL Annexin V antibody conjugated by FITC for 20 min. Then, cells were washed with 200 μL binding buffer and 5 μL of Propidium Iodide Staining Solution. Cells were analyzed by flow cytometry immediately.

### Exosome isolation

Exosomes in the medium were isolated by differential centrifugation. Cells and other fragments were removed by centrifugation at 300 g and 3000 g respectively, and then, the other larger vesicles were removed by centrifuging the supernatant at 10,000 g for 40 min. Finally, exosomes were collected when centrifuged the supernatant at 110,000 g for 80 min, and resuspended in PBS. The exosomes were imaged by transmission electron microscopy (Thermo Scientific, USA).

### Immunohistochemistry (IHC)

Paraffin-embedded gastric cancer specimens were sectioned and fixed on slides. Anti-NRP2 antibody (1:200, Abcam) was used to stain the protein. And horseradish peroxidase (HRP)-conjugated goat anti-rabbit IgG (ZSGB-BIO, China) was used as the secondary antibody. Staining intensity and distribution were assessed by experienced pathologists.

### Lentivirus infection

The lentivirus-containing GFP-puro-shRNA-NRP2 or empty plasmids were purchased from Genechemo (Shanghai, China). And, lentivirus concentrate was added to CAFs for 12 h (MOI 1:10). Green fluorescence was typically visualized after 2–3 days. And, CAFs were selected by 1 μg/mL puromycin for 1–2 passages.

### Statistics

Experiments mentioned in our study were performed in triplicate and presented as the mean value ± SD. Results were analyzed using *t* test or one-way ANOVA in SPSS. Log-rank test was applied to compare survival between groups, and Cox proportional hazard ratio model was used to find prognostic factors from clinicopathological parameters. Statistically significant was considered when *P* < 0.05. *indicates *P* < 0.05; **indicates *P* < 0.01; and ***indicates *P* < 0.001.

## Results

### Isolation of primary fibroblasts from human gastric cancer tissues

**Fig. 1:**
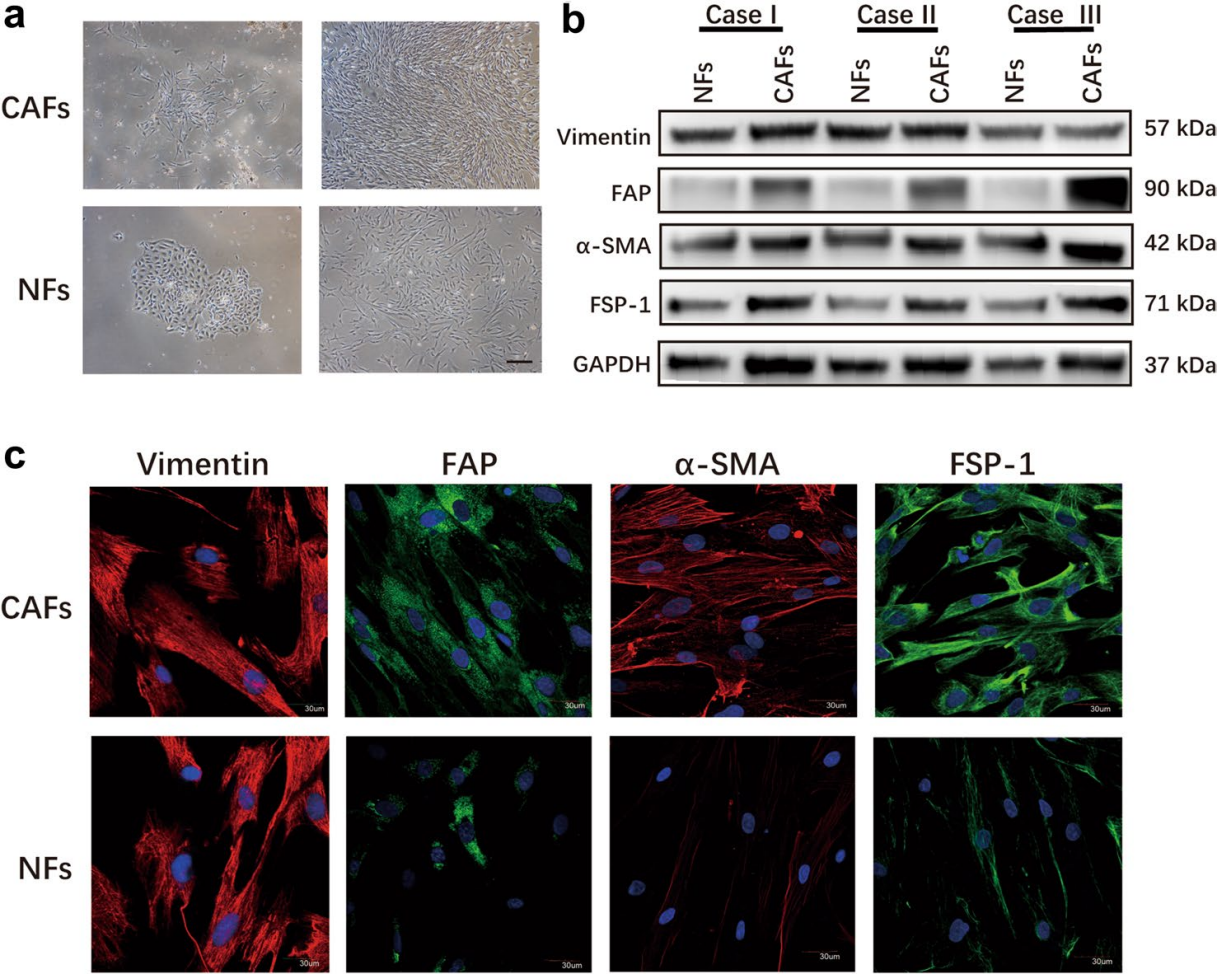
Identification of fibroblasts. **a** The morphology of CAFs and NFs under microscope. Scale bar: 1 mm. **b** Western blot analysis of Vimentin, α-SMA, FAP, and FSP1 protein levels in CAFs and NFs. **c** Immunofluorescence staining for Vimentin, α-SMA, FAP, and FSP1 expression of CAFs and NFs. Scale bar: 30 μm

The primary CAFs were isolated from the tumor tissues, and NFs were acquired from the paired normal gastric tissues. CAFs appeared as radial spokes with sharp edges at first, whereas NFs arranged like paving stones. The proliferation rate of CAFs was significantly faster than that of NFs, and they eventually became whorled and storiform, which was difficult to distinguish by visual observation (Fig. 1a). Immunofluorescence staining assays and western blot revealed that the expressions of FAP, α-SMA, and FSP1 in CAFs were higher than that of NFs, indicating that the primary CAFs were activated fibroblasts. As a specific marker of stromal cells, vimentin is equally expressed in CAFs and paired NFs (Fig. 1b and c).

### CAFs induce chemoresistance of gastric cancer cells via exosomes

**Fig. 2:**
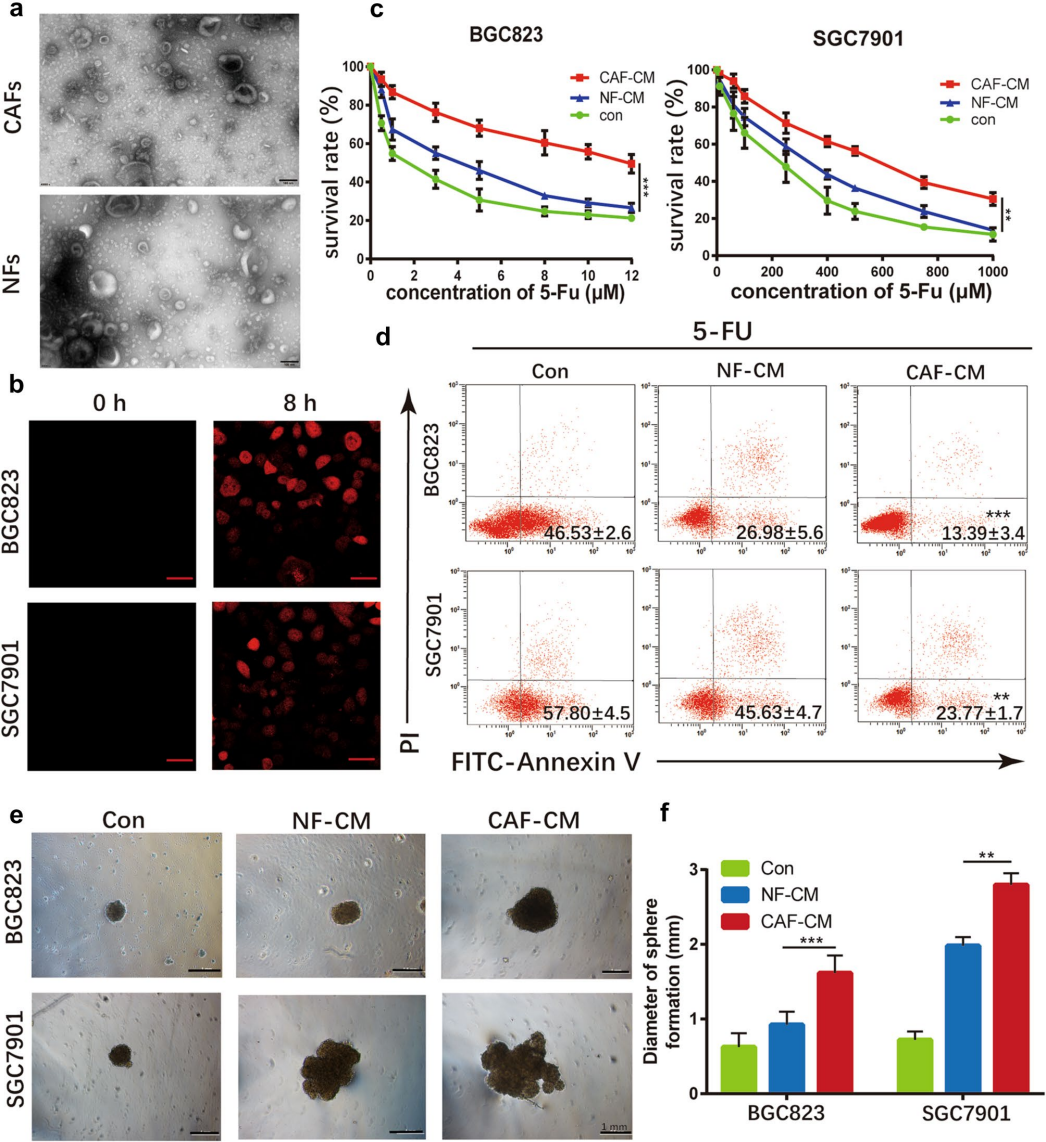
CAF-derived exosomes confer 5-FU resistance of gastric cancer cells. **a** TEM images of exosomes isolated from primary CAFs. Scale bar: 100 nm. **b** Fluorescence microscope images of the internalization of fluorescently labeled CAF exosomes in BGC823 and SGC7901 cells. Scale bar: 20 μm. **c** The survival rates of 5-FU on BGC823 and SGC7901 cells cultured by CAFs-CM, NFs-CM, and normal medium, respectively. **d** Apoptosis after 5-FU treatment in BGC823 and SGC7901 cells cultured by CAFs-CM, NFs-CM, and normal medium, respectively. The proportion of Annexin V^+^/PI^−^ (early apoptosis) and Annexin V^+^/PI^+^ (late apoptosis) cells was shown. **e** Representative images of coculture sphere formation. Scale bar: 1 mm. **f** Diameter quantitative analysis of sphere formation

It is well known that exosomes can transfer bioactive particles such as proteins, lncRNAs, and miRNAs from CAFs to cancer cells, affecting the activities of recipient cells [27–32]. Primarily, exosomes were isolated from the conditioned medium (CM) of CAFs and NFs by ultra-centrifugation. The diameters of exosomes were chiefly 40–150 nm (Fig. 2a). To determine whether exosomes from CAFs were taken up by gastric cancer cells, gastric cancer cells were cultured in CAF-CM, in which exosomes were labeled with PKH-26. After 8 h, the red fluorescent dyes were observed in both SGC7901 and BGC823 cells (Fig. 2b), indicating that CAF-derived exosomes can efficiently fuse with cancer cells.

To confirm the function of CAFs in chemotherapy resistance, we cultured SGC7901 and BGC823 cells with CAF-CM, NF-CM, and normal medium, respectively. When challenged the tumor cells with 5-fluorouracil (5-FU), survival of the tumor cells was significantly enhanced upon cultured with CAF-CM, rather than NF-CM or normal medium (Fig. 2c). Consistently, CAF-CM protected SGC7901 and BGC823 from chemotherapy-induced apoptosis effectively (Fig. 2d). To further investigate the interactions between fibroblasts and cancer cells, CAFs and NFs were co-cultured with gastric cancer cells separately. And, we adopted the 3D-coculture technique to imitate in vivo conditions (Fig. 2e). Agreement with previous experiments, the diameter of CAFs-tumor sphere was significantly larger than NFs, when challenged by 5-FU (Fig. 2f).

### Analysis of differentially expressed genes between CAFs and NFs

**Fig. 3:**
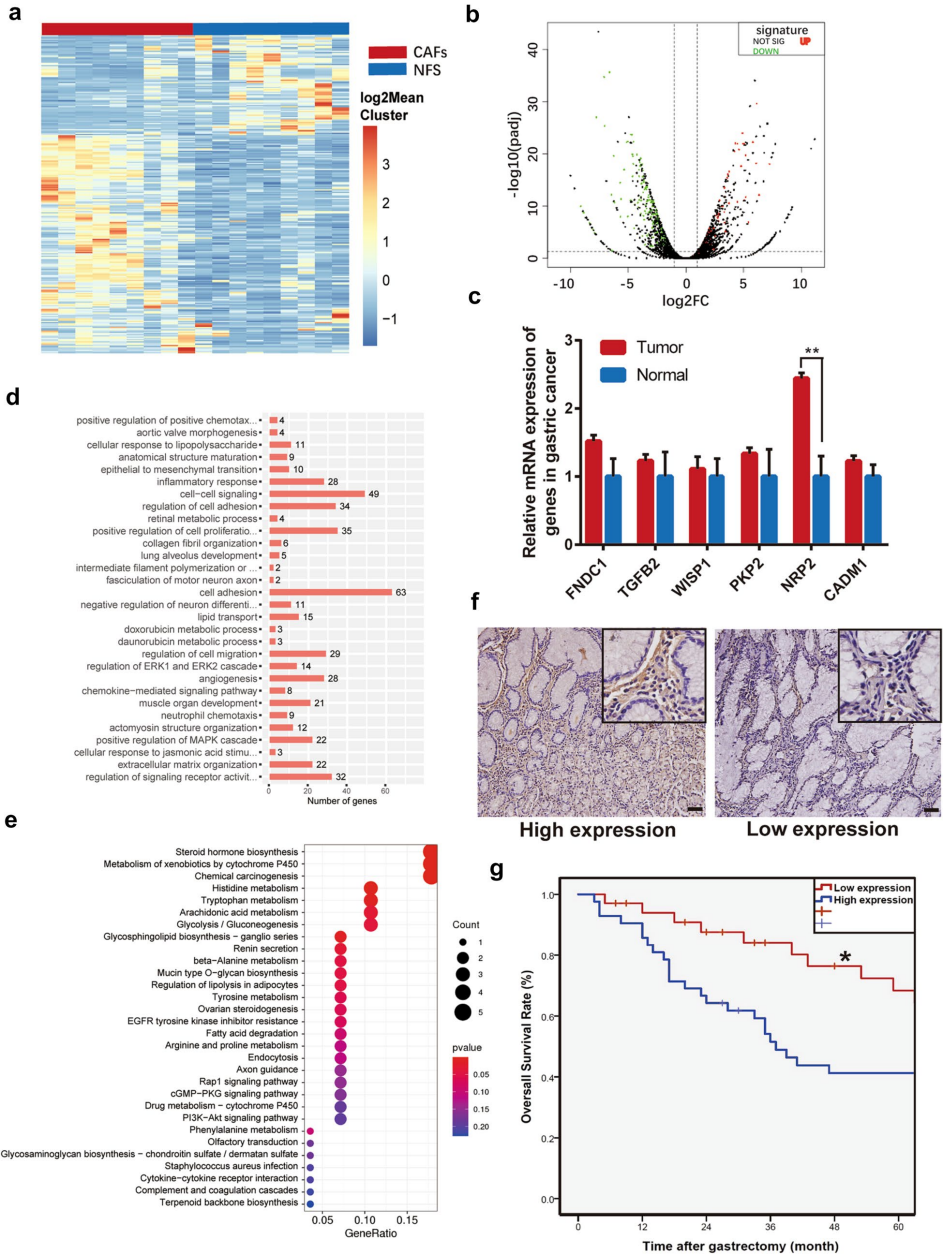
Analysis of differentially expressed genes between CAFs and NFs **a** Heatmap for differentially expressed mRNAs in CAFs compared with NFs. **b** Volcano plots of downregulated and upregulated mRNAs. **c** Expression of six candidates in gastric cancer and normal gastric tissue. **d** Gene oncology terms analysis. **e** KEGG pathway enrichment analysis. **f** Representative IHC staining images of NRP2 in gastric cancer tissues (*n* = 72). Scale bar: 1 mm. **g** High expression of NRP2 in gastric cancer CAFs predicts worse overall survival

To systematically identify mRNAs related to gastric cancer progression, 18 samples from 9 advanced gastric cancer patients were sequenced using RNA-sequence (Fig. 3a and b). Gene Ontology (GO) and Kyoto Encyclopedia of Genes and Genomes (KEGG) pathway enrichment analysis shows that genes in the cluster are mainly related to intercellular signaling, cell adhesion, and carcinogenesis (Fig. 3c and d). And, 37 highly expressed mRNAs were found in over 50% of the nine CAFs strains. According to the *P* value, fold change, and oncological function of these genes, six candidate genes were picked up for further research. Finally, we detected the expression of these six highly expressed mRNAs in CAFs by qPCR (Fig. 3e). And, we found that NRP2 was the major different transcript between CAFs and NFs. According to The Cancer Genome Atlas (TCGA) database, NRP2 was upregulated in other types of tumors compared with normal cells (Supplementary Fig. 1). These results indicate that NRP2 commonly acts as an oncogene. Furthermore, we checked the expression of NRP2 in 72 gastric cancer specimens by immunohistochemistry (IHC) assay (Fig. 3f). Analyzed by Kaplan–Meier survival curves, low expression levels of NRP2 in CAFs were associated with better overall survival (OS) of gastric cancer patients (Fig. 3g). Clinicopathological characteristics were further assessed by Cox proportional hazard ratio model, and we found that higher expression of NRP2 in CAFs was an independent prognostic factor in gastric cancer patients (Supplementary file 1). Subsequently, we decided to choose NRP2 for further confirmation.

### NRP2 affects 5-FU sensitivity by SDF-1 in gastric cancer

**Fig. 4:**
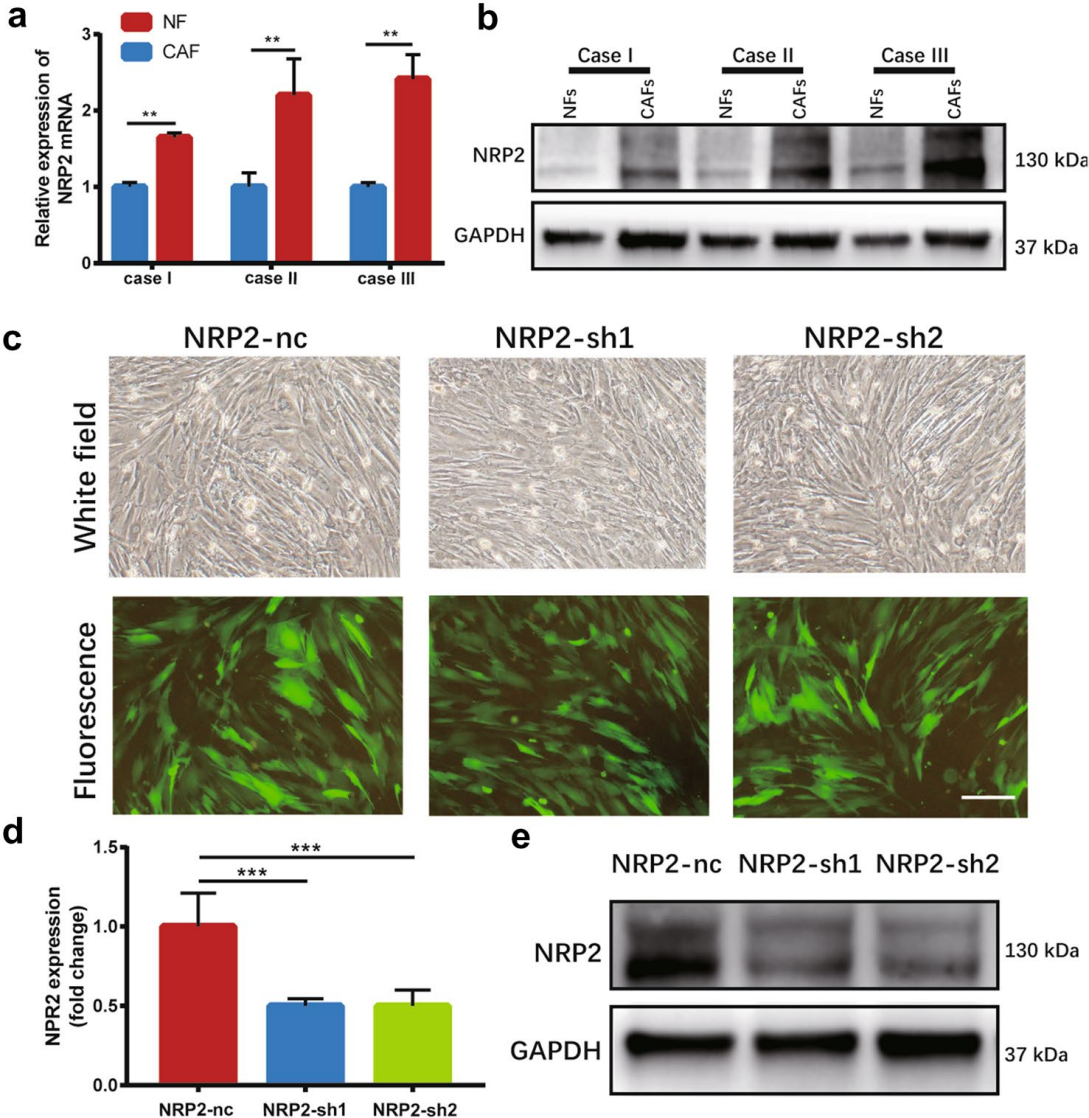
Reduced NRP2 affects 5-FU sensitivity for gastric cancer. **a** Relative mRNA expression of NRP2 in CAFs and NFs. **b** Protein expression of NRP2 in CAFs and NFs. **c** The morphology of CAFs-nc and CAFs-sh cells as observed via bright field and corresponding fluorescence field. Scale bar: 1 mm. **d** and **e** qPCR and western blot results showed significant inhibition of NRP2 expression in CAFs-sh cells compared with that in CAFs-nc cells

To confirm the upregulation of NRP2 in CAFs, we used qPCR and western blot to examine the expression of NRP2 in CAFs and NFs. Both mRNA and protein levels of NRP2 in CAFs were significantly higher than those in NFs (Fig. 4a and b). Then, NRP2 was knocked down with short hairpin RNA (shRNA) by lentivirus infection to investigate whether NRP2 contributes to 5-FU resistance in gastric cancer. The green fluorescence indicated a high infection rate (Fig. 4c). qPCR and western blot showed that the expression of NRP2 was inhibited significantly in the NRP2-sh CAFs (Fig. 4d and e).

**Fig. 5:**
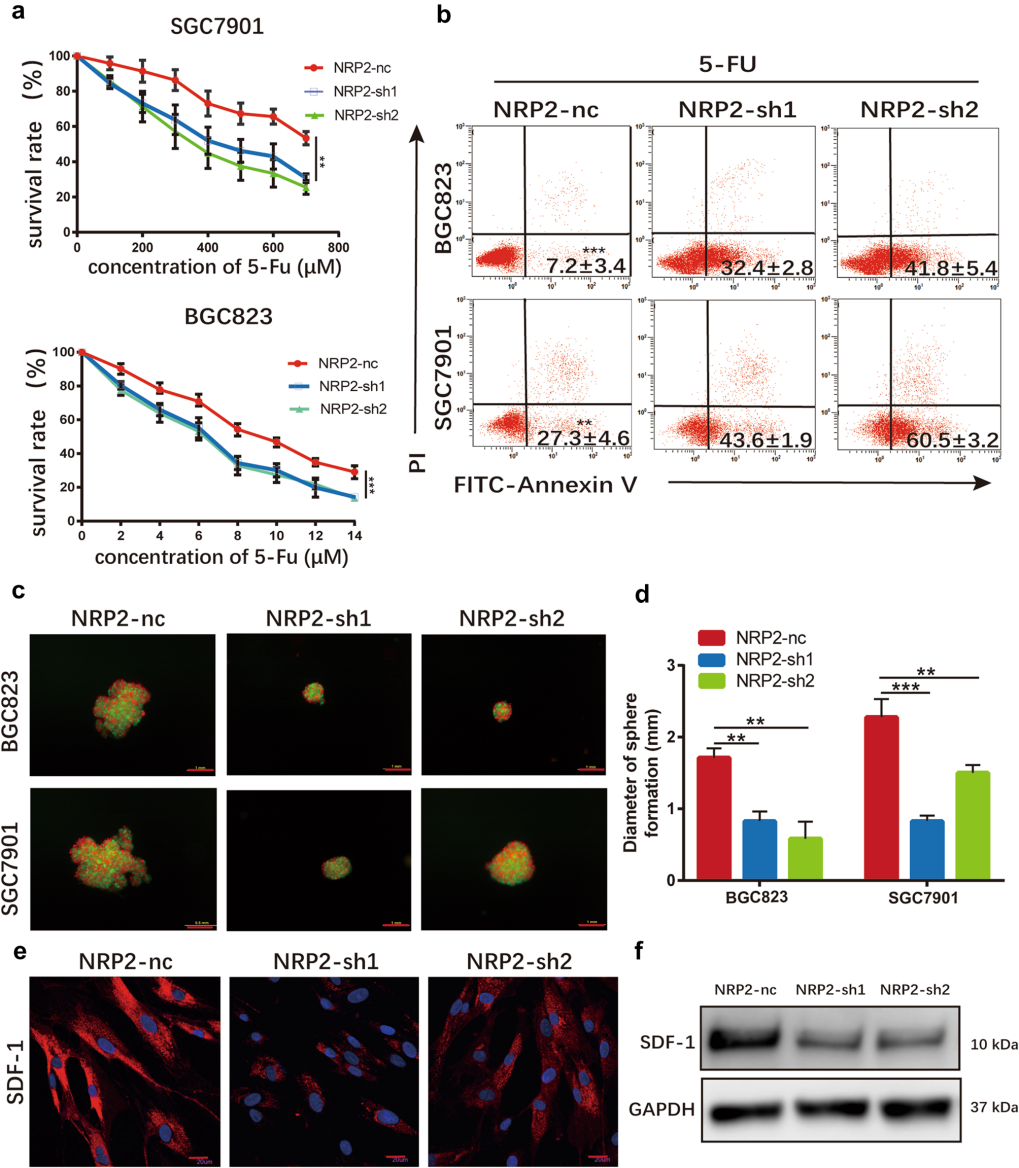
Reduced NRP2 affect 5-FU sensitivity for gastric cancer. **a** The survival rates of 5-FU on BGC823 and SGC7901 cells cultured by CM from CAFs-nc, CAFs-sh1, and CAFs-sh2 respectively. **b** Apoptosis after 5-FU treatment in BGC823 and SGC7901 cells cultured by CM from CAFs-nc, CAFs-sh1, and CAFs-sh2 respectively. The proportion of Annexin V^+^ cells was shown. **c** Representative images of coculture sphere formation. Gastric cancer cells and CAFs were fluorescently labeled by mScarlet and GFP, respectively. Scale bar: 1 mm. **d** Diameter quantitative analysis of sphere formation. **e** Immunofluorescence staining for SDF-1 expression of CAFs. Scale bar: 30 μm. **f** Western blot results showed the decreased expression of SDF-1 followed the knockdown of NRP2

We cultured SGC7901 and BGC823 cells with supernatant obtained from infected CAFs cells or normal CAFs cells, respectively. When challenged by 5-FU, we found that downregulation of NRP2 eradicated the protection of CAFs on tumor cells against cytotoxic agents (Fig. 5a). And, chemotherapy-induced apoptosis was enhanced because of the silencing of NRP2 compared with control cell strain (Fig. 5b). To simulate the stereoscopic growth pattern of gastric cancer and fibroblasts, 3D techniques were applied to set up the coculture model. For the sake of observation, we transferred the red fluorescence mScarlet into gastric cancer cell lines with a nontargeting sequence. Infected CAFs were co-cultured with SGC7901 and BGC823 cells and challenged by 5-FU, and images were obtained through fluorescence microscopy (Fig. 5c). And the average diameters of NRP2-knockdown tumor spheres were obviously less than that of control tumor spheres (Fig. 5d).

It has been reported that stromal cell-derived factor-1 (SDF-1), also called CXCL12, is the predominant transducer in VEGF/NRP2 signaling [33, 34]. And, SDF-1 is mainly derived from stromal cells; thus, we hypothesized that SDF-1 is the major downstream effector in CAFs. Expression levels of SDF-1 were quantified by immunofluorescence and western blot. We found that expression levels of SDF-1 were decreased when NRP2 was knocked down in CAFs (Fig. 5e and f). Together, these findings illustrated that NRP2 probably affect 5-FU sensitivity by downstream effector SDF-1 in gastric cancer.

### YAP/TAZ is necessary for NRP2 to protect cancer cells from 5-FU induced DNA damage

**Fig. 6:**
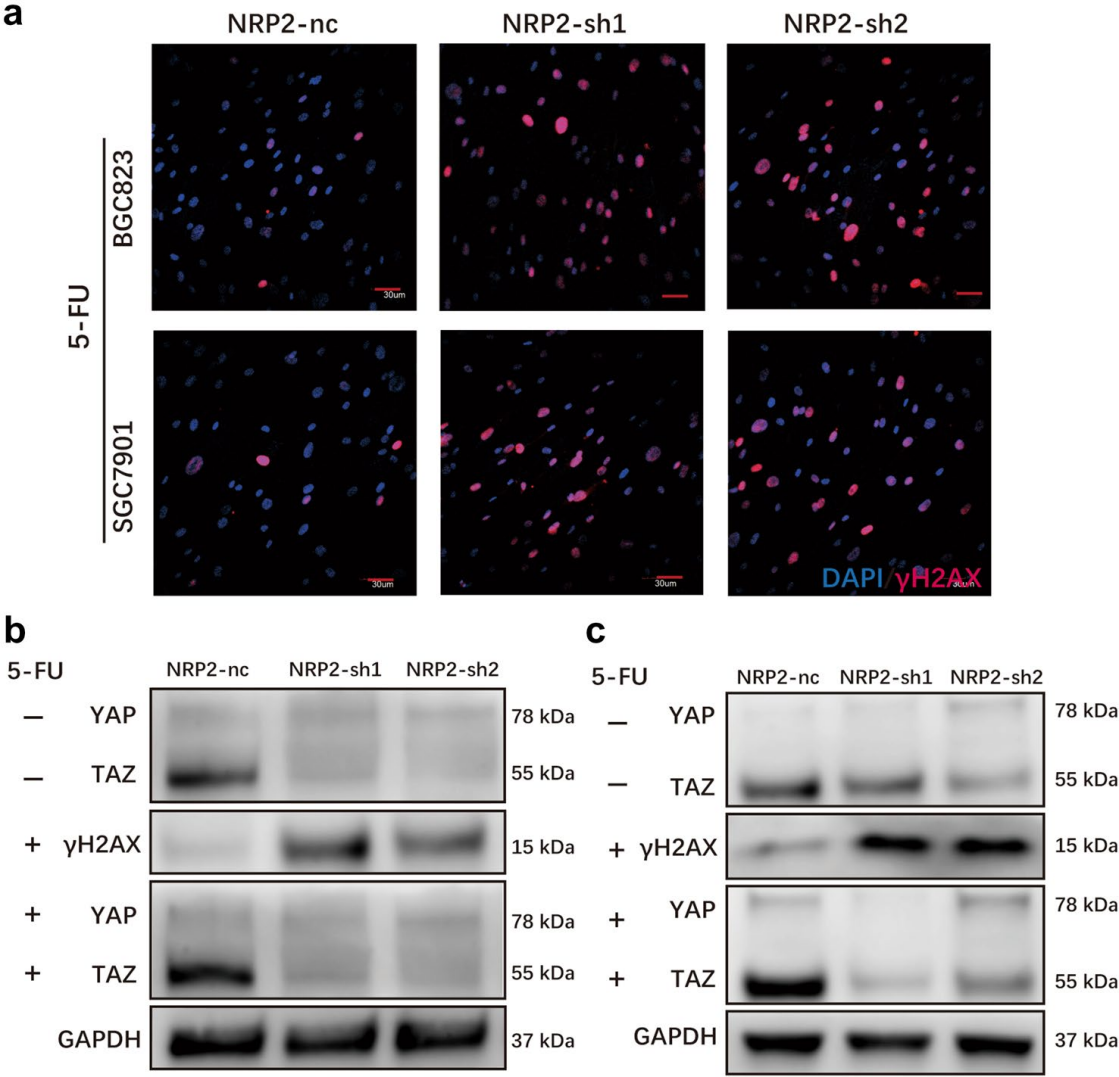
YAP/TAZ are necessary for NRP2 protection from 5-FU induced DAN damage **a** Representative immunofluorescence staining of γH2AX in BGC823 and SGC7901 cells cultured by CM from CAFs-nc, CAFs-sh1, and CAFs-sh2 when treated with 5-FU. **b** and **c** Expression of γH2AX and YAP/TAZ in BGC823 (**b**) and SGC7901 (**c**) cells challenged by 5-FU, and gastric cancer cells were cultured by corresponding CM

It is known that 5-FU is a cell cycle-specific agent and can induce DNA damage. To confirm whether the resistance of gastric cancer cells to 5-FU was mediated by enhanced DNA damage repair, we measured the expression of γH2AX to examine the DNA damage in the cells. γH2AX is a marker of DNA damage, and it locates in the cell nucleus. For this purpose, we cultivated gastric cancer cells with CM collected from NRP2-sh CAFs and NRP2-nc CAFs, respectively. When challenged by 5-FU, we observed that the knockdown of NRP2 resulted in an increase of DNA damage in comparison with control cells (Fig. 6a). These results indicated that the high expression of NRP2 in CAFs promotes the resistance of gastric cancer cells to DNA-damaging chemotherapy via enhanced DNA damage repair.

NRP2 is proved to be the receptor of VEGF, and multiple evidences showed that the Hippo pathway transducers YAP and TAZ are critical downstream effectors of VEGF signaling, while they are also crucial factors in the process of DNA damage [26, 35]. Therefore, we hypothesized that NRP2 promotes 5-FU resistance through the activation of YAP/TAZ in gastric cancer cells. To verify our hypothesis, we assessed the expression of YAP/TAZ in NRP2-sh CAF-CM-treated cancer cells and found that YAP/TAZ significantly diminished, compared with NRP2-nc CAF-CM-treated cancer cells. The expression of YAP/TAZ was consistent with the previous results when challenged by 5-FU (Fig. 6b and c). These data provide evidence that NRP2 protects cancer cells from DNA damage by a mechanism that involves YAP/TAZ activation in gastric cancer cells.

## Discussion

Chemoresistance is the major challenge to the treatment of gastric cancers. The mechanism that related to chemoresistance is complex and has not been comprehensively understood. Therefore, resistance to chemotherapy sets up a barrier between cancer patients and oncologists [36]. Chemotherapy is the predominant method of postoperative therapy for advanced gastric cancers, and 5-FU is the first-line chemotherapeutic drugs. Coincidentally, resistance to 5-FU is becoming more and more serious in gastric cancer therapy [37]. Resistance to chemotherapy is generally related to cancer cell DNA damage repair and alterations of the particles that affecting cell apoptosis [38, 39]. To overcome this barrier, there is an urgent need to explore the molecular mechanism behind the chemoresistance of gastric cancer.

It is known that CAFs are the dominant stromal cells in the TME. Up to now, the origins of CAFs are still unknown, and some investigators found that they come from mesenchymal stem cells (MSCs) [40]. MSCs are also important cellular components in TME. Gastric cancer-derived MSCs have been proven to promote cancer cells progression by secreting IL-8, microRNA, and PDGF-DD [41–43]. Meanwhile, MSCs communicate with other immune cells like neutrophils to affect gastric cancer cells [40]. And the correlation between CAFs and MSCs still need further investigation. Emerging evidence has demonstrated that CAFs can affect cancer chemoresistance through multiple interactions [44]. In the present study, we found that gastric cancer cells had a significantly higher survival rate when cultured in CAFs supernatant or coculture with CAFs and challenged by 5-FU. And, we hypothesized that the difference between CAFs and NFs may play a crucial part in the chemoresistance of gastric cancer.

To further investigate the difference between CAFs and NFs, we collected nine pairs of gastric cancer tissues and matched para-carcinoma tissues from surgical specimens. Primary fibroblasts were isolated and cultured for study. To explore the transcriptome, we extract mRNA from paired fibroblasts for RNA-sequencing. Based on high-throughput sequencing technology and bioinformatics analysis, we discovered and characterized an expanded landscape of fibroblasts transcriptomic data, which have never been reported. After analysis, we found that NRP2 was recurrently upregulated in nine CAF strains compared with matched NFs.

As has been reported, NRP2 participates in cancer cell metastasis via lymphatic invasion, and blocking NRP2 could repress metastasis [45–47]. NRP2 also plays a vital role in cancer cell chemoresistance [25, 26]. However, the functions of NRP2 in CAFs have never been studied. Primarily, we verified that the expression of NRP2 was obviously abundant in CAFs than NFs both in RNA and protein levels. Furthermore, IHC assay of gastric cancer specimens illustrated that low expression levels of NRP2 in CAFs were associated with better overall survival of gastric cancer patients. And higher expression of NRP2 in CAFs was an independent prognostic risk factor. Then, we knocked down the NRP2 in CAFs and found that the effect of protecting cancer cells from chemotherapy diminished. And, we creatively adopted 3D coculture to simulate the real interactions between CAFs and tumor cells. The average diameters of NRP2-sh tumor spheres were noticeably less than that of control spheres when challenged by 5-FU. What happened below the surface in cancer cells truly fascinates us. It is known that 5-FU can cause DNA damage in cancer cells. To confirm whether the resistance to 5-FU in gastric cancer cells was associated with DNA damage repair, we introduced a marker of DNA damage, γH2AX. We found that the γH2AX was patently lower in cancer cells cultured by NRP2-nc CAF-CM than NRP2-sh CAF-CM. And, we were given a hint that DNA damage repair enhanced in these cancer cells on account of the normal expression of NRP2 in CAFs. These results illustrated that the normal expression of NRP2 in CAFs could irritate itself to secrete particular molecules under the cross-talk between CAFs and cancer cells, and these molecules can be taken up by cancer cells, which may help the process of DNA damage repair. The stromal cell-derived factor-1 (SDF-1) is the predominant effector of VEGF/NRP2 signaling [33, 34]. And, we found that the expression levels of SDF-1 were decreased following NRP2-knockdown, which indicated that NRP2 probably affect the secretion of SDF-1. The SDF-1 may be taken by cancer cells and furtherly impact on the resistance of 5-FU. It has been reported that the Hippo pathway transducers YAP and TAZ are critical downstream effectors of VEGF signaling, while are also crucial factors in DNA damage [26, 35]. In the present study, the expression of YAP/TAZ was decreased when cancer cells are cultured by NRP2-sh CAF-CM compared with control cells. And the result was repeated when challenged by 5-FU.

As mentioned above, NRP2 is a receptor of VEGF. The results of the present study found that the VEGF/NRP2 signaling in CAFs can promote chemoresistance in gastric cancer. Importantly, we also demonstrated that this mechanism is mediated by the YAP/TAZ activation in cancer cells. These findings integrate the VEGF/NRP2 signaling in CAFs and the Hippo pathway in cancer cells into a unified mechanism that accounts for their therapy resistance. And the SDF-1 may be the bridge from CAFs to cancer cells, thus influences the response of tumor cells to cytotoxic agents. We firmly believe that the mechanisms are far more complicated than the present study. And whether targeting NRP2 of CAFs represents a precise therapy needs our further investigation.

## Conclusion

The present study indicated that CAFs within gastric cancers promote chemoresistance through the expression of NRP2. The secretion of SDF-1 that mediated by VEGF/NRP2 signaling in CAFs and the activation of Hippo pathway in cancer cells in large part participated in this project.

## Supplementary Information

Below is the link to the electronic supplementary material.

Supplementary file1 (DOCX 18 KB)

Supplementary Fig. 1 NRP2 expression was upregulated in other kinds of tumors compared with the matched normal cells (TIF 69556 KB)

## Data Availability

The materials and data that we used in our study are availability from the corresponding author if necessary.
