# Supplementary material for: CAF promotes chemoresistance through NRP2 in gastric cancer
Source: Gastric Cancer. 2021 Nov 26;25(3):503–14. doi: 10.1007/s10120-021-01270-w (PMC9013334; doi:10.1007/s10120-021-01270-w)
Supplement: Supplementary file 1 — Supplementary file1 (DOCX 18 KB) [file 10120_2021_1270_MOESM1_ESM.docx]

**Supplementary Table 1** Clinical characteristics of included patients in the present study

| **No.** | **Age**  **(years)** | **Gender** | **Height**  **(cm)** | **Weight**  **(Kg)** | **Clinical stage** | **Preoperative treatment** | **Pattern of gastrectomy** | **Preoperative pathology** | **Differentiation** |
| --- | --- | --- | --- | --- | --- | --- | --- | --- | --- |
| **I.** | 67 | male | 172 | 60 | T3N1 | none | subtotal | adenocarcinoma | moderate |
| **II.** | 61 | female | 160 | 60 | T2N1 | none | total | adenocarcinoma | poor |
| **III.** | 72 | male | 160 | 70 | T3Nx | none | total | adenocarcinoma | poor |
| **IV.** | 60 | male | 170 | 55 | T3N1 | none | total | adenocarcinoma | moderate |
| **V.** | 74 | male | 170 | 68 | T3N0 | none | subtotal | adenocarcinoma | poor |
| **VI.** | 68 | female | 163 | 62 | T3N0 | none | subtotal | adenocarcinoma | moderate |
| **VII.** | 57 | male | 165 | 58 | T3N2 | none | subtotal | adenocarcinoma | poor |
| **VIII.** | 69 | male | 175 | 68 | T2N0 | none | subtotal | adenocarcinoma | poor |
| **IX.** | 73 | male | 165 | 62 | T3N0 | none | total | adenocarcinoma | moderate |

| **Variables** | **Univariate analysis** | | | **Multivariate analysis** | | |
| --- | --- | --- | --- | --- | --- | --- |
|  | **HR** | **95% CI** | ***P* value** | **HR** | **95% CI** | ***P* value** |
| **Age**  ≤65 *vs.* >65 | 0.761 | 0.405-1.429 | 0.396 |  |  |  |
| **Gender**  Female vs. male | 0.832 | 0.385-1.798 | 0.636 |  |  |  |
| **Tumor size**  ≤5cm *vs.* >5cm | 0.508 | 0.285-1.161 | 0.077 |  |  |  |
| **pT**  T1-3 *vs.* T4 | 0.402 | 0.173-0.937 | 0.043 |  |  |  |
| **pN**  N0 *vs.* N+ | 0.236 | 0.057-0.981 | 0.013 | 0.379 | 0.032-0.984 | 0.033 |
| **Stage**  I-III *vs.* IV | 0.098 | 0.012-0.803 | 0.029 | 0.232 | 0.067-0.802 | 0.038 |
| **NRP2 expression**  Low *vs.* High | 0.387 | 0.252-0.941 | 0.032 | 0.489 | 0.368-1.632 | 0.046 |

**Supplementary Table 2** Univariate and multivariate analysis of NRP2 expression in gastric cancer
